# The helminth product, ES-62, protects against airway inflammation by resetting the Th cell phenotype

**DOI:** 10.1016/j.ijpara.2012.12.001

**Published:** 2013-03

**Authors:** Justyna Rzepecka, Ivonne Siebeke, Jennifer C. Coltherd, Dorothy E. Kean, Christina N. Steiger, Lamyaa Al-Riyami, Charles McSharry, Margaret M. Harnett, William Harnett

**Affiliations:** a Strathclyde Institute of Pharmacy and Biomedical Sciences, University of Strathclyde, 161 Cathedral Street, Glasgow G4 0RE, UK; b Institute of Infection, Immunity and Inflammation, University of Glasgow, Glasgow G12 8TA, UK

**Keywords:** Asthma, Airway inflammation, Parasitic helminth, ES-62, IFNγ, IL-4, IL-17, Neutrophil

## Abstract

We previously demonstrated inhibition of ovalbumin-induced allergic airway hyper-responsiveness in the mouse using ES-62, a phosphorylcholine-containing glycoprotein secreted by the filarial nematode, *Acanthocheilonema viteae*. This inhibition correlated with ES-62-induced mast cell desensitisation, although the degree to which this reflected direct targeting of mast cells remained unclear as suppression of the Th2 phenotype of the inflammatory response, as measured by eosinophilia and IL-4 levels in the lungs, was also observed. We now show that inhibition of the lung Th2 phenotype is reflected in ex vivo analyses of draining lymph node recall cultures and accompanied by a decrease in the serum levels of total and ovalbumin-specific IgE. Moreover, ES-62 also suppresses the lung infiltration by neutrophils that is associated with severe asthma and is generally refractory to conventional anti-inflammatory therapies, including steroids. Protection against Th2-associated airway inflammation does not reflect induction of regulatory T cell responses (there is no increased IL-10 or Foxp3 expression) but rather a switch in polarisation towards increased Tbet expression and IFNγ production. This ES-62-driven switch in the Th1/Th2 balance is accompanied by decreased IL-17 responses, a finding in line with reports that IFNγ and IL-17 are counter-regulatory. Consistent with ES-62 mediating its effects via IFNγ-mediated suppression of pathogenic Th2/Th17 responses, we found that neutralising anti-IFNγ antibodies blocked protection against airway inflammation in terms of pro-inflammatory cell infiltration, particularly by neutrophils, and lung pathology. Collectively, these studies indicate that ES-62, or more likely small molecule analogues, could have therapeutic potential in asthma, in particular for those subtypes of patients (e.g. smokers, steroid-resistant) who are refractory to current treatments.

## Graphical abstract

**Graphical abstract:**

**Figure:**
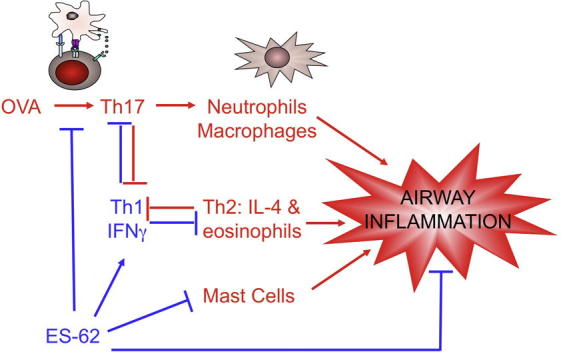

**Highlights:**

► A worm-derived product, ES-62, protects against allergic airway inflammation induced by ovalbumin in mice. ► Protection is associated with resetting of the Th1/Th2 balance and correlates with suppression of Th17 responses. ► The study provides important information on the mechanism of action of a parasitic helminth-derived immunomodulator. ► The immunomodulator offers novel and safe therapeutic potential in the treatment of allergic diseases.

## 1 Introduction

The incidence of asthma has doubled throughout developed countries in recent decades (Umetsu et al., 2002) such that it is now highly prevalent in many parts of the world. The Hygiene Hypothesis aims to explain such an increased incidence as being a consequence of improved sanitation and hygiene in modernised societies, with the lack of infections in childhood preventing us from developing an immune system that responds appropriately to environmental antigens (Strachan, 1989). This has led to the idea that the common exposure to parasite infections in developing countries protects individuals from atopic asthma, prevalent in developed countries. This concept has been particularly investigated with respect to helminth parasites and although the situation is by no means clear-cut (Leonardi-Bee et al., 2006), evidence can certainly be found to support a protective role for various platyhelminths and nematodes against asthma and allergy in animal models (Harnett and Harnett, 2010).

Asthma is a chronic pulmonary disease characterised by airway inflammation, acute reversible airway obstruction, bronchial hyper-responsiveness and high levels of serum IgE (Holgate, 1999). The inflammation exhibited in asthma is generally accepted as being associated with a Th2 phenotype immune response with infiltrates of mast cells, eosinophils, macrophages and Th2 lymphocytes and their cytokines such as IL-5, IL-4 and IL-13 being present in increased amounts in the lungs (Busse and Lemanske, 2001). IL-5 induces eosinophils to differentiate from bone marrow progenitor cells and to move to the lung and expand to form a local IL-4-producing innate cell population. This further amplifies the Th2 cytokine environment (Chen et al., 2004), resulting in increased IgE production (Bossie et al., 1987) which, when found on the surface of mast cells, is centrally involved in the pathogenesis of asthma (Busse and Lemanske, 2001). Collectively, these responses ultimately result in airway hyper-responsiveness (AHR) and remodelling.

ES-62 is an immunomodulatory phosphorylcholine (PC)-containing glycoprotein discovered in the rodent filarial nematode *Acanthocheilonema viteae* (reviewed in Harnett and Harnett, 2010). ES-62 possesses a number of anti-inflammatory properties (Whelan et al., 2000; Goodridge et al., 2001; Harnett and Harnett, 2010) and consistent with this, we have shown that prophylactic ES-62 treatment is protective in a mouse model of Th1/Th17-mediated inflammatory autoimmune disease, collagen-induced arthritis (McInnes et al., 2003). Similarly, and consistent with the proposal that helminth infections may protect from allergic inflammatory diseases, we found that the anti-inflammatory actions of ES-62 extended to inhibition of inflammation exhibited in the lungs in the murine ovalbumin (OVA)-induced model of allergic asthma (Melendez et al., 2007). These data suggest that ES-62 has therapeutic potential in the treatment of asthma and hence it is important to elucidate its mechanism of action. Prophylactic exposure to ES-62 reduced disease severity and progression as indicated by histological analysis of lung pathology and whole-body plethysmography determination of airway hyper-reactivity and remodelling. The protection observed in mice correlated with ES-62-induced desensitisation of mast cells, which have been implicated in airway remodelling (Carter and Bradding, 2011; Gilfillan and Beaven, 2011), and also with suppression of the Th2 phenotype of airway inflammation, the latter as evidenced by reduced eosinophilia and IL-4 levels in the lungs (Melendez et al., 2007). Therefore, we investigated the mechanisms by which ES-62 acts to suppress the Th2-mediated parameters of OVA-induced airway disease.

## 2 Materials and methods

### 2.1 Mice and reagents

Six to 8 week old female BALB/c mice were purchased from Harlan Olac (Bicester, UK) and maintained at the Universities of Glasgow and Strathclyde, UK. All procedures were conducted in accordance with Home Office, UK animal guidelines and with the approval of the local ethical committees. Purified, endotoxin-free ES-62 from the rodent filarial nematode, *A. viteae,* was produced as described previously (Wilson et al., 2003). Neutralising anti-IFNγ antibodies were purified using Protein G Sepharose, Fast Flow (Sigma Aldrich, Dorset, UK) from cell line XMG1.6, which was a kind gift from Prof. Richard Grencis at the University of Manchester, UK. The IgG isotype control (rat IgG_1_) was obtained from Bio X Cell (West Lebanon, NH, USA).

### 2.2 Allergic airway model

Allergic airway inflammation was induced as described previously (McKay et al., 2004). Briefly, 6–8 week old female BALB/c mice were sensitised to OVA by i.p. injection of 100 μg of OVA in 200 μl of 1% alum (Alhydrogel; Brenntag Biosector, Fredriksund, Denmark) on days 0 and 14. On day 14, mice were challenged by the intranasal (i.n.) route with 50 μg of OVA in 30 μl of PBS (endotoxin-free, Lonza, Slough, UK) after anaesthesia was induced with isoflurane. On days 25, 26 and 27 mice were anaesthetised and re-challenged i.n. with 50 μg of OVA in 30 μl of PBS. Control mice received PBS in place of OVA. Mice were subjected to euthanasia on day 28 by lethal i.p. injection of avertin (1,1,1-tribromoethanol) dissolved in iso-amyl alcohol and diluted 1 in 40 in PBS, and bronchoalveolar lavage (BAL) and lung histology were performed as described previously (Melendez et al., 2007). There were four experimental groups denoted: PBS (control), ES-62, OVA and OVA + ES-62. ES-62 and OVA + ES-62 mice received 2 μg of ES-62 in 100 μl of PBS, by s.c. injection in the scruff of the neck on days −2, 12, 25 and 27. Mice in the control and OVA groups received PBS on these days. The concentration of ES-62 used has been shown to be likely to give serum levels equivalent to those found for PC-containing molecules during filarial nematode infection of humans (Lal et al., 1987; Wilson et al., 2003). For the studies using neutralising anti-IFNγ antibodies, mice in OVA and OVA + ES-62 groups were i.p. injected with either 150 μg of anti-IFNγ or isotype control IgG (both endotoxin free) in 150 μl of PBS on days 1, 15 and 26. The control IgG antibody had no significant effect on any of the OVA responses tested (results not shown).

### 2.3 Ex vivo lymph node cultures

Lungs were dissected and the peribronchial draining lymph nodes (DLNs; thoracic) harvested. DLN cells were cultured in RPMI 1640 medium at 10^6^ cells/ml with 10% FBS, penicillin (100 U/ml), streptomycin (100 μg/ml), l-glutamine (2 mM), 2-mercaptoethanol (50 μM), 1% non-essential amino acids and sodium pyruvate (1 mM) (all from Gibco Life Technologies, Paisley, UK). Cells were cultured in medium alone or in medium containing antigen (OVA at 500 μg/ml) or concanavalin A (ConA, 3 μg/ml) for the 72 h culture period. For proliferation analysis, cells were pulsed with [^3^H] thymidine (0.5 μCi/well; Amersham Pharmacia Biotech, Little Chalfont, UK) for the last 4 h of culture. For cytokine analysis, samples were centrifuged at the end of the culture period for 5 min at 400*g* and the supernatant removed and stored at −20 °C until further analysis.

### 2.4 Cytokine and antibody detection

Cytokines in culture supernatants and serum were analysed using ELISA kits (BD Biosciences, Oxford, UK apart from that for IL-17: BioLegend Ltd., Cambridge, UK) or by employing the Luminex system (also used for all other cytokines, chemokines and growth factors referred to) with a 20-Multiplex inflammatory cytokine kit (Biosource, Invitrogen, Paisley, UK) according to manufacturers’ instructions. The lower limit of detection for all cytokines apart from IL-17 (15 pg/ml) was 20 pg/ml for ELISA and 5 pg/ml for IL-4 and IL-13, 10 pg/ml for IL-5, 15 pg/ml for IL-10 and 1 pg/ml for IL-17 and IFNγ by Luminex. Bio-plex Manager software with five parametric curve fitting was used for data analysis (Bio-Rad Laboratories Inc., USA). Total IgE and OVA-specific IgG1, IgG2a and IgE levels were measured in serum by ELISA (BD Biosciences, UK) following the manufacturer’s instructions using Maxisorp ELISA plates (Nunc, Roskilde, Denmark).

### 2.5 In vitro dendritic cell (DC)-T cell cocultures

Bone marrow-derived dendritic cells (bmDCs) were prepared as described previously (Goodridge et al., 2004, 2005). Briefly, bone marrow cells were cultured in RPMI-1640 complete medium containing 2 mM l-glutamine, 50 U/ml of penicillin, 50 μg/ml of streptomycin, 10% FCS (Invitrogen) supplemented with 10% conditioned medium generated by the GM-CSF-secreting X63 myeloma cell line and 50 μM 2-mercaptoethanol, for 6 days at 37 °C/5% CO_2_, with fresh medium supplied on day 4. On day 7, the loosely adherent immature bmDCs were harvested, pulsed with peptide antigen (Ag) (OVA_332–339_) and cocultured (2.5 × 10^4^ cells) with OVA-specific CD4^+^CD62L^+^ T cells (2 × 10^5^ cells) from DO.11.10/BALB/c mice, purified using CD4^+^ T cell negative selection and enrichment for naïve CD62L^high^ cells (Miltenyi Biotec Ltd., UK) as described previously (Whelan et al., 2000; Marshall et al., 2005). Cells were cocultured in a total volume of 1 ml of RPMI 1640 medium supplemented with 10% FBS, penicillin (100 U/ml), streptomycin (100 μg/ml), l-glutamine (2 mM), 2-mercaptoethanol (50 μM), 1% non-essential amino acids and sodium pyruvate (1 mM) in 24 well plates for 72 h at 37 °C and 5% CO_2_. Cell supernatants were then removed and stored at −20 °C for future cytokine analysis.

### 2.6 Analysis of intracellular cytokine production and transcription factor expression

Intracellular cytokine production was assessed using the Cell Fixation and Permeabilisation solutions and protocols provided by eBioscience (Hatfield, UK) as described previously (Pineda et al., 2012). Briefly, DLN cells were stimulated with RPMI-1640 medium or 500 ng/ml of phorbol 12-myristate 13-acetate (PMA) plus 500 ng/ml of ionomycin and 1xBrefeldin A (eBioscience, UK) for 4 h at 37 °C with 5% CO_2_. Cells were stained with Fixable Viability Dye eFluor 780 (eBioscience) prior to staining for cell surface phenotypic markers using anti-CD4-PerCP or anti-CD8-FITC (both BD Pharmingen, UK). The cells were then fixed and permeabilised before staining with the relevant cytokine-specific APC-conjugated (anti-IL-10: BD Pharmingen; anti-IFNγ and anti-IL-17: Biolegend) or PE-conjugated (anti-IL-4, BD Pharmingen) antibody. Intracellular Foxp3 and RORγt expression in DLN cells was similarly analysed using the APC-conjugated Foxp3- and PE-conjugated RORγt-specific antibodies, and staining buffer sets and protocols from eBioscience.

### 2.7 Quantitative real time PCR (qRT-PCR)

qRT-PCR procedures were carried out according to the manufacturer’s instructions (Applied Biosystems, Carlsbad CA, USA). IL-4, IL-17, IFNγ and glyceraldehyde 3-phosphate dehydrogenase (GAPDH) levels were tested using Applied Biosystems assay kits, Mm00445259_m1, Mm00439619_m1, Mm01168134_m1 and Mm99999915_g1. All samples were examined in triplicate and data analysed by StepOne software using the comparative C_T_ (ΔΔC_T_) (Applied Biosystems) with values for samples being normalised to the reference reporter GAPDH. Lungs stored in RNAlater (Qiagen, Crawley, UK) were transferred into RTL plus buffer (Qiagen) supplemented with β-mercaptoethanol and homogenised with the TissueRuptor (Qiagen). The homogenate was then centrifuged for 3 min at maximum speed and the lysate was used to extract RNA using a RNAeasy plus kit (Qiagen). Up to 1 μg of RNA was reverse transcribed using a High Capacity cDNA Reverse Transcription Kit (Applied Biosystems) and 50 ng of RNA was used per qRT-PCR.

### 2.8 Analysis of lineage-specific transcription factor expression in tissue sections

Briefly, tissue sections (6 μm) were incubated in acetone for 20 min, air dried and rehydrated with PBS containing 1% goat serum before being incubated in blocking reagent containing 6% BSA, 10% goat serum and 1% Fc-block. For staining of transcription factors, 0.1% (v/v) TritonX (Sigma) was used whilst for IgE, 0.5% saponin was added to all buffers. Avidin solution (Vector Laboratories Ltd., Peterborough, UK) was added for 15 min to block unmasked endogenous biotin, then biotin solution (Vector Laboratories) was added to block excess avidin. Purified or biotinylated primary antibodies and isotype controls were added either for 1 h at room temperature (surface marker) or overnight at 4 °C (intracellular staining). Fluorochrome conjugated secondary antibodies or streptavidin conjugates were added for 1 h before mounting using Vectashield. Images (20× magnification) were captured using a Hammamatsu camera and analysed using Openlab imaging software (Improvision, Coventry, UK). Quantitative laser scanning cytometry (LSC) analysis was performed as described previously (Adams et al., 2004; Morton et al., 2007) where standard cell contours were set to determine individual transcription factor-positive T cells. Tissue maps were generated from these data and the number and percentage of transcription factor-positive T cells located within the T cell area were determined using WinCyte software (CompuCyte Corp, Essex, UK).

### 2.9 Statistical analysis

Statistical analysis was determined by *t*-test and ANOVA or Kruskal–Wallis (non-parametric data) with Bonferroni or Dunn post-tests, respectively, as appropriate. *P* values of ^∗^ ⩽0.05, ^∗∗^ ⩽0.01 and ^∗∗∗^ ⩽0.001 were used to denote significance.

## 3 Results

### 3.1 ES-62 suppresses infiltration of a heterogeneous population of inflammatory cells into the lungs of mice undergoing OVA-induced airway inflammation

**Fig. 1:**
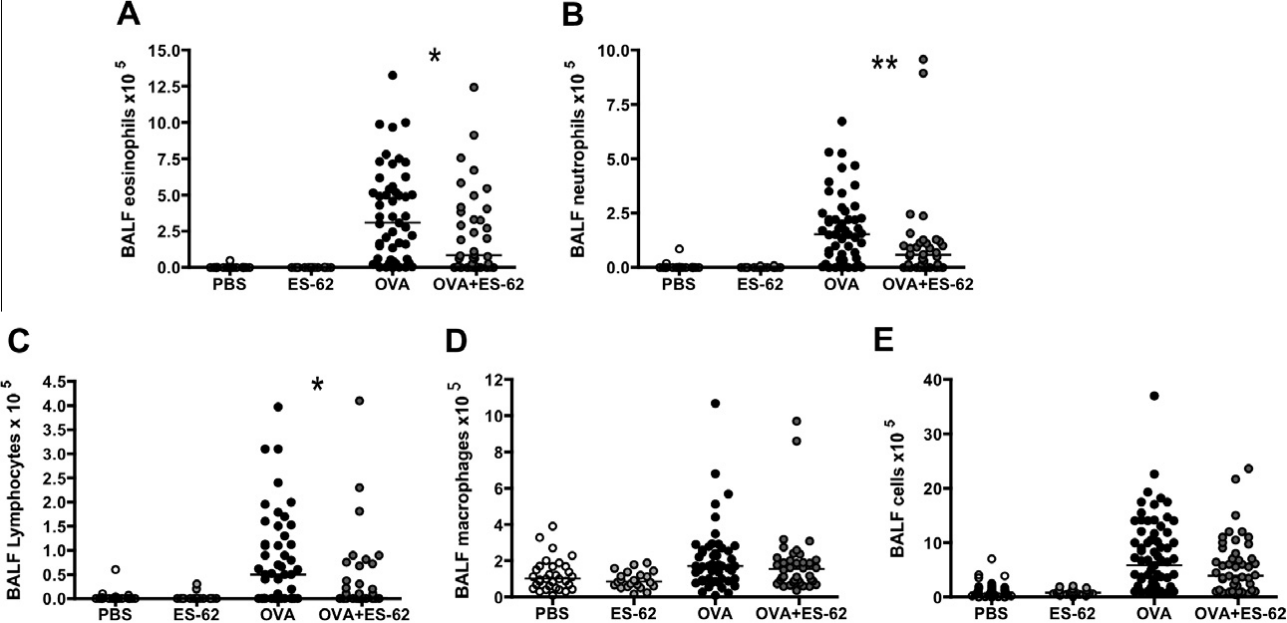
The phosphorylcholine-containing glycoprotein, ES-62, inhibits eosinophil, neutrophil and lymphocyte infiltration of the lungs in the murine ovalbumin (OVA)-induced airway inflammation model. Differential eosinophil (A), neutrophil (B), lymphocyte (C), macrophage (D) and total (E) bronchoalveolar lavage fluid (BALF) cell counts in individual mice from each of the four treatment groups where, for A–D, *n* = 32 for PBS*; n* = 22 for ES-62*; n* = 51 for OVA and *n* = 38 for OVA + ES-62 groups and for E, *n* = 42 for PBS*; n* = 24 for ES-62*; n* = 59 for OVA and *n* = 44 for OVA + ES-62 groups. The bar represents the median value of the group and ^∗^*P* ⩽ 0.05 or ^∗∗^*P* ⩽ 0.01 for OVA compared with OVA + ES-62 groups.

Prophylactic exposure to ES-62 in vivo reduces lung pathology and, specifically, eosinophilia in the mouse OVA-induced airway inflammation model, thereby protecting against OVA-induced hypersensitivity (Melendez et al., 2007). These results are now confirmed and extended by our showing that in addition to preventing eosinophilic inflammation (Fig. 1A), ES-62 also significantly prevented infiltration of the lungs by neutrophils (Fig. 1B) and lymphocytes (Fig. 1C). ES-62 also tended to reduce the numbers of macrophages and total cells in the BAL fluid (BALF) but this did not reach statistical significance (Fig. 1D, E).

### 3.2 ES-62 suppression of lung inflammation is associated with reduced local Th2 cytokine responses

**Fig. 2:**
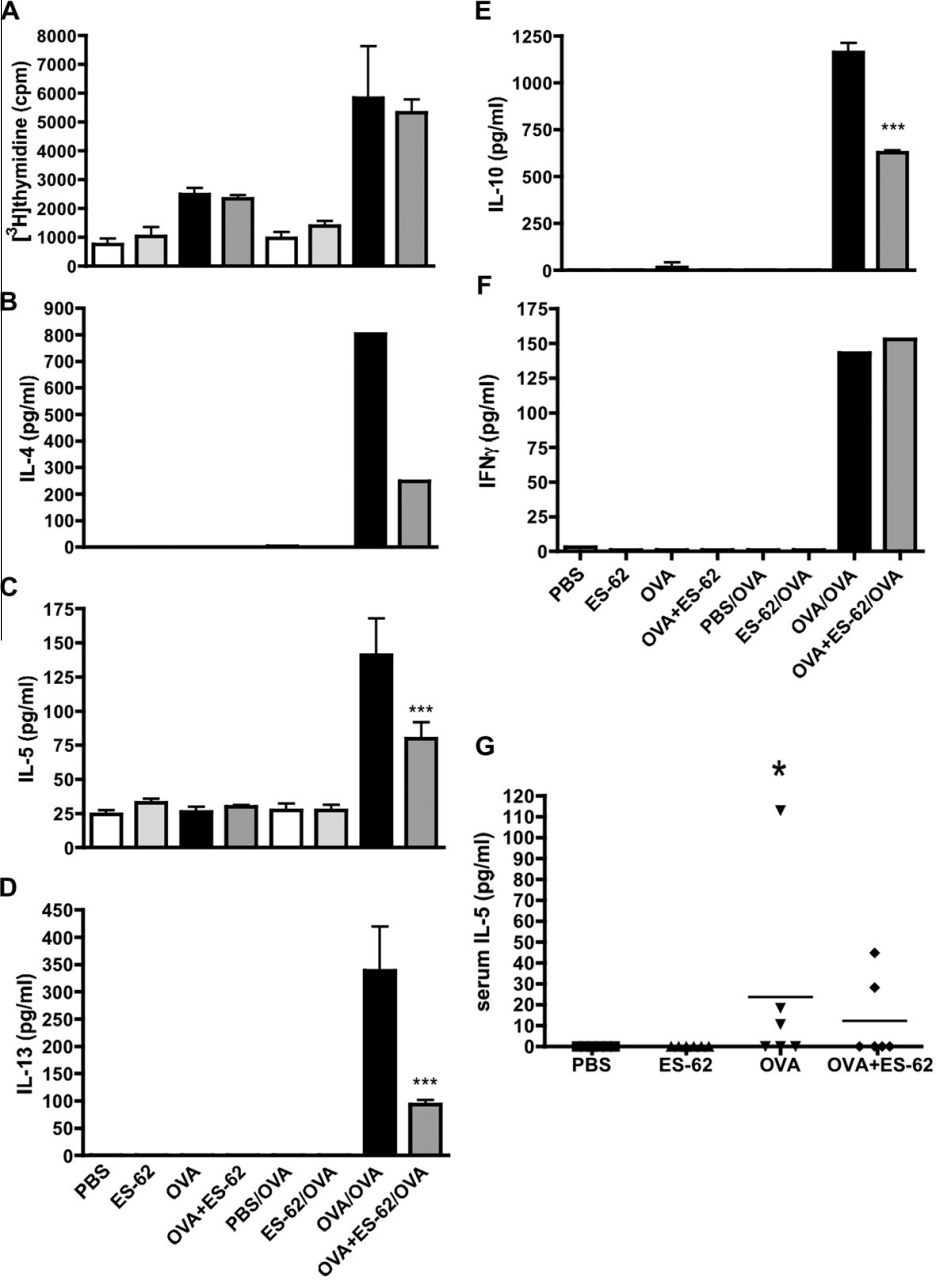
In vivo treatment with the phosphorylcholine-containing glycoprotein, ES-62, inhibits draining lymph node (DLN) cell antigen-specific Th2 cytokine production but not antigen-specific proliferation ex vivo. Lung DLN (thoracic) cells from individual mice within each in vivo treatment group were pooled and cultured with medium alone or ovalbumin (OVA) (500 μg/ml) for 72 h. Cell proliferation was measured by [^3^H] thymidine uptake and data (A) are expressed as mean ± S.D. (*n* = 3 replicate cultures) and are from one experiment representative of two. Culture supernatants IL-4 (B), IL-5 (C), IL-13 (D), IL-10 (E) and IFNγ (F) were measured for each group and data are expressed as mean concentrations ± S.D., *n* = 3 replicate cultures, where ^∗∗∗^*P* ⩽ 0.001, apart from IL-4 and IFNγ which were measured as duplicate samples by Luminex, and represent single data sets representative of at least two independent experiments apart from IL-13 which was only measured in a single experiment to corroborate the decrease in the very low levels of IL-4 observed in that model. (G) The mean values of triplicate serum (obtained day 28) values from individual mice (*n* = 6) are shown where the bar represents the mean of the group and the OVA, but not OVA + ES-62, group shows significantly elevated levels of IL-5 (^∗^*P* ⩽ 0.05) relative to the PBS and ES-62 control groups.

To address whether such protection reflects immunomodulation of polarised Th responses during OVA-induced airway inflammation, the effect of in vivo exposure to ES-62 on subsequent peribronchial DLN recall responses to OVA ex vivo was determined. Firstly, cell proliferative responses were analysed and it was noted that whilst DLN cells from mice that had received the airway inflammation protocol (OVA and OVA + ES-62 groups) showed elevated basal levels of proliferation relative to those from PBS (control) or ES-62 groups (^∗∗∗^*P* ⩽ 0.001; Fig. 2A), stimulation with OVA ex vivo strongly enhanced this proliferation (^∗^*P* ⩽ 0.05 for OVA groups and ^∗∗∗^*P* ⩽ 0.001 for OVA + ES-62 groups) but there were no significant differences found between the responses of the OVA and OVA *+* ES-62 groups. ES-62 treatment alone had little effect on cytokine production in DLN cells (Fig. 2B–F), however, and consistent with OVA-induced inflammation reflecting a Th2 phenotype, IL-4, IL-5, IL-13 and IL-10 production was detected in cells from mice undergoing OVA-induced airway inflammation and this was found to be reduced in cells from such mice exposed to ES-62 in vivo (Fig. 2B–E). Conversely, exposure to ES-62 did not suppress OVA-specific IFNγ production (Fig. 2F). To support these findings of suppression of Th2 responses, we undertook an experiment measuring cytokine levels in serum samples and whilst the low levels of IL-5 detected in mice from the OVA group were significantly elevated relative to mice treated with PBS or ES-62 alone, the levels in the OVA + ES-62 group were not significantly different from the control groups (Fig. 2G). However, serum levels of IL-4, IL-10, IL-13 and IFNγ in all groups were found to be below the detection level of the assay in use. It was also not possible to detect eotaxin. The chemokines, MCP-1, MIG (CXCL9) and MIP-1α, and the growth factor, FGF, which have been implicated in the inflammation and increased angiogenesis associated with remodelling in asthma (Linden, 2003; Kolls and Linden, 2004; Havaux et al., 2005; Bosse et al., 2006; Feltis et al., 2006) were detected; however, serum levels of these inflammatory mediators were not found to be significantly different amongst the four treatment groups (results not shown). The Th2-suppressive effects of ES-62 on OVA-treated mice were Ag-specific because they were not mirrored in the mitogen (ConA)-induced proliferation and cytokine responses of DLN cells (results not shown).

### 3.3 ES-62 treatment inhibits IgE production

**Fig. 3:**
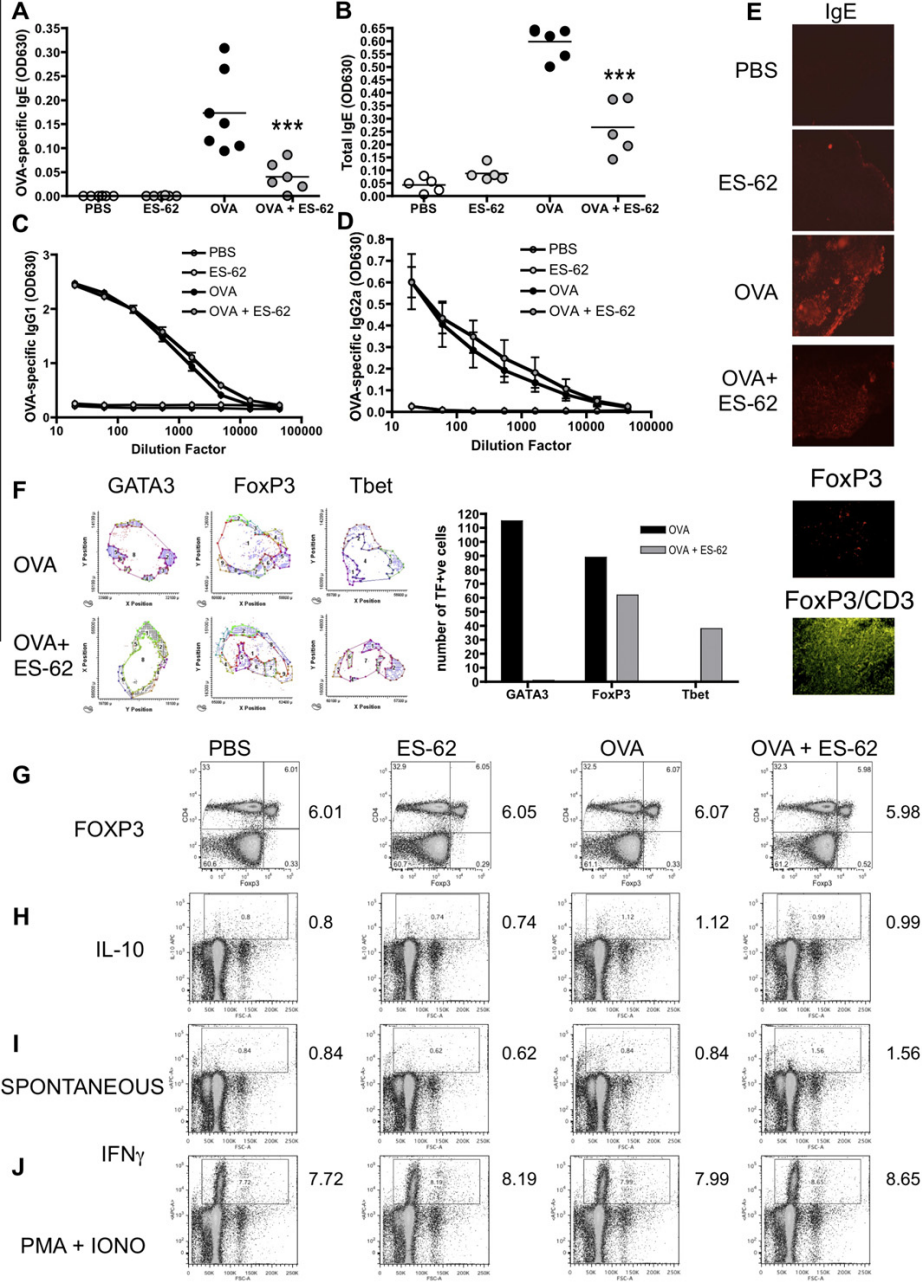
Exposure to the phosphorylcholine-containing glycoprotein, ES-62, in vivo does not modulate ovalbumin (OVA)-induced elevation of IgG1 and IgG2a but inhibits IgE and suppresses Th2 cells. Serum samples from each mouse were obtained on day 28. Samples were analysed by ELISA for OVA-specific IgE (A) and total IgE (B), at 1/80 and 1/200 dilutions, respectively, and OVA-specific IgG1 (C) and IgG2a (D) over the full titration range where data for each group are presented as the mean ± S.E.M. (*n* = 6 mice/group) and ^∗∗∗^*P* ⩽ 0.001, OVA + ES-62 compared with the OVA group. The data are from one experiment representative of two. In E, draining lymph nodes (DLN) cells from each of the four treatment groups were stained for expression of IgE. (F) DLN sections from OVA and OVA + ES-62 groups were stained for B220^+^ B cells, CD3^+^ T cells and for the indicated transcription factor (either GATA3, Foxp3 or Tbet) and the transcription factor positive cells were quantified within the T cell regions. Exemplar tissue maps from a single experiment of DLNs from OVA and OVA + ES-62 groups displaying the relevant distribution of such transcription factor positive cells (red) within the T cell paracortical region (white) surrounded by B220^+^ B cells (blue) and their quantitation of numbers of the relevant transcription factor positive T cells by laser scanning cytometry (LSC) are shown, together with an exemplar relocated image of a portion of the section from the OVA group where Foxp3 is stained in red and CD3, green (F). In an independent experiment, the proportions of Foxp3-expressing unstimulated CD4^+^ T cells from the DLN of pooled mice from the four treatment groups are shown, with the percentage of cells in the Foxp3^+^CD4^+^ quadrant annotated to the right of the plots (G). Pooled DLN cells from the four treatment groups were cultured with medium (I) or phorbol 12-myristate 13-acetate (PMA) plus ionomycin (IONO) (H, J) in the presence of Brefeldin A before staining for intracellular expression of IL-10 (H) or IFNγ (I, J) prior to FACS analysis: cytokine expression (*y*-axis) was plotted against side scatter (*x*-axis) and the percentage of DLN cells in the cytokine positive gate is shown and annotated to the right of the plots. (J) The Mean Fluorescence Intensity (MFI) values for IFNγ production for the four treatment groups are PBS: 24,701; ES-62: 21,915; OVA: 23,439 and OVA + ES-62: 27,596. (For interpretation of the references to colour in this figure legend, the reader is referred to the web version of this article.)

As antibodies, especially those of the signature Th2 isotype, IgE, have great influence in directing and enhancing the inflammatory immune response in allergic airway disease (Busse and Neaville, 2001; Busse and Lemanske, 2001), it was next determined whether the anti-inflammatory action of ES-62 was reflected by a change in the antibody profile in vivo. Thus, sera from all groups of mice were analysed in vitro for total IgE and OVA-specific IgG1, IgG2a and IgE content. Whilst IgE levels were elevated in the OVA, relative to the control and ES-62 groups (Fig. 3A, B), this elevation in antibody production was significantly inhibited by prophylactic treatment with ES-62 (OVA + ES-62 group) (*P* ⩽ 0.001 for both total and OVA-specific IgE). However, analysis of sera IgG demonstrated that levels of OVA-specific IgG1 and IgG2a antibody isotypes in mice that had received the OVA administration protocol were not influenced by exposure to ES-62 (Fig. 3C, D). Consistent with the observed reduction in serum IgE levels, lower levels of IgE expression could be detected in DLN cells from OVA + ES-62, relative to OVA, mice (Fig. 3E).

### 3.4 Exposure to ES-62 in vivo modulates the Th1/Th2 balance in OVA-induced airway inflammation

To investigate whether ES-62 was suppressing the development of Th2 responses in OVA-induced airway inflammation by induction of regulatory T (Treg) cells, DLN tissue sections were stained for expression of the signature Treg, Th1 and Th2 transcription factors, Foxp3, Tbet and GATA3, respectively (Fig. 3F), and imaged and quantitatively analysed by LSC (Adams et al., 2004; Morton et al., 2007). Consistent with the observed inhibition of Th2 responses, the number of cells expressing GATA3 was reduced essentially to zero following ES-62 exposure but this did not appear to reflect suppression by Treg cells as the number of Foxp3 positive cells was also slightly reduced in the DLN cells from ES-62-treated model mice (Fig. 3F). These data were corroborated by flow cytometric analysis of DLN cells from an independent experiment that showed no substantial differences in the levels of Foxp3^+^CD4^+^Tregs amongst the different treatment groups (Fig. 3G) or in production of the Treg effector cytokine, IL-10, by DLN cells (Fig. 3H). By contrast, however, there was an appearance of cells expressing Tbet in DLN sections from the ES-62-treated OVA model mice (Fig. 3F) suggesting that ES-62 might act by resetting the Th1/Th2 balance in OVA-induced airway inflammation. Likewise, data from an independent experiment showed that exposure to ES-62 in vivo resulted in increased levels of spontaneous (Fig. 3I) IFNγ production by DLN cells from mice undergoing OVA-induced airway inflammation, with the OVA + ES-62 group exhibiting an approximately twofold increase in the small proportion of DLN cells producing IFNγ, relative to those from OVA mice, which exhibited comparable levels to those of naïve animals. Consistent with our previous findings that exposure to ES-62 alone induces a weak Th2 phenotype in naïve healthy mice (Whelan et al., 2000; Marshall et al., 2005), the lowest levels of spontaneous IFNγ-producing cells were observed in DLNs from the ES-62 group. Moreover, the OVA + ES-62 group contained the highest percentage of IFNγ-producing cells (and the highest expression of IFNγ within cells) following stimulation with PMA plus ionomycin (Fig. 3J) whilst the lowest proportion of such cells was found in the DLN from the control (PBS) group of mice.

### 3.5 Neutralising anti-IFNγ antibodies inhibit the protection against airway inflammation afforded by ES-62

**Fig. 4:**
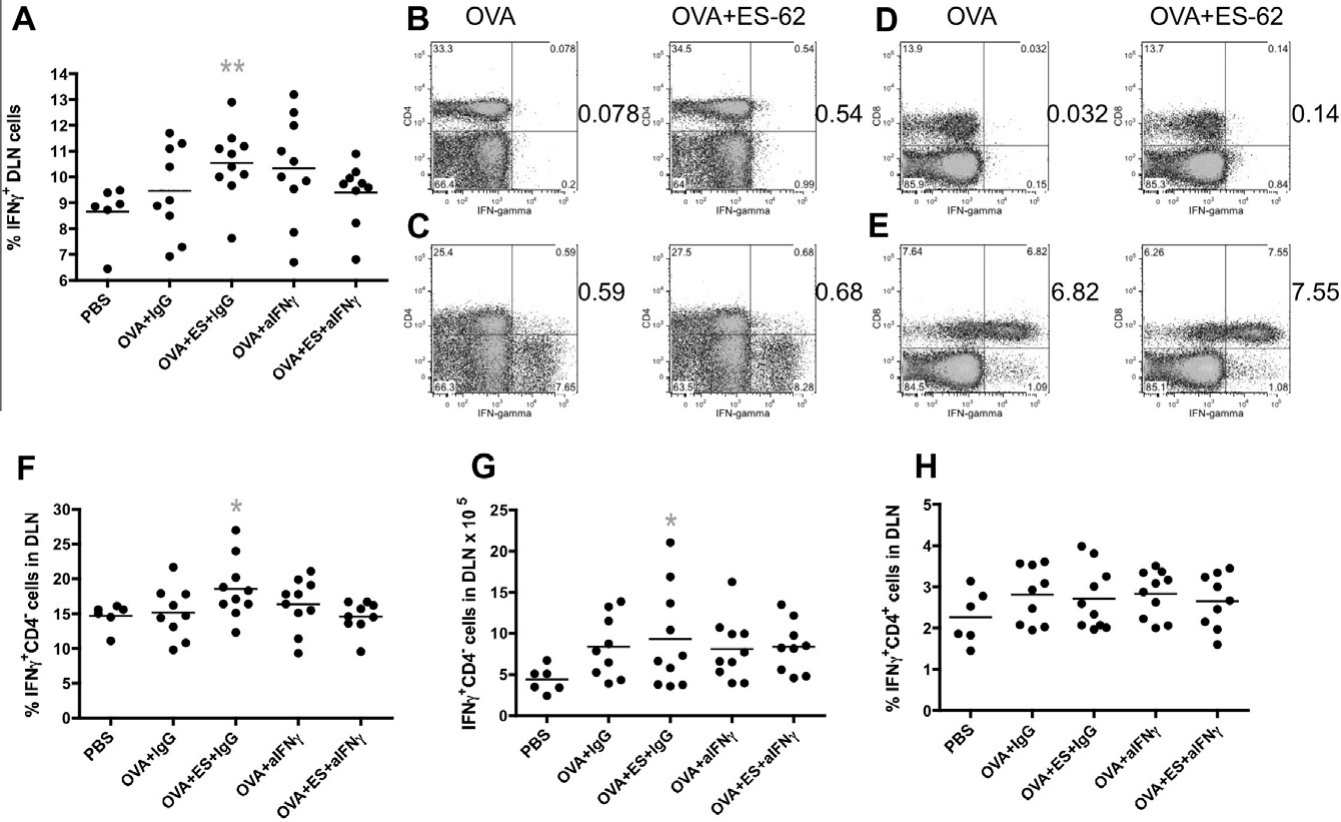
Exposure to the phosphorylcholine-containing glycoprotein, ES-62, in vivo increases CD4 and CD8 IFNγ responses during murine ovalbumin (OVA)-induced airway inflammation. (A) The proportion of draining lymph node (DLN) cells expressing IFNγ following stimulation with phorbol 12-myristate 13-acetate (PMA) plus ionomycin in the indicated treatment groups by individual mice (PBS, *n* = 6; OVA + IgG, *n* = 9; OVA + ES-62 + IgG, *n* = 10; OVA + anti-IFNγ, *n* = 10 and OVA + ES-62 + anti-IFNγ, *n* = 9; pooled from two independent experiments). The bar represents the mean of the group and the OVA + ES-62 + IgG, but not OVA + IgG, group is significantly different from the PBS group (grey ^∗∗^*P* ⩽ 0.01). In an independent experiment, the cytokine expression (*y*-axis) by cells from pooled DLN from the indicated groups was analysed and the proportions of IFNγ-producing cells in the lymphocyte gate of DLN cells from mice from the OVA and OVA + ES-62 groups that were CD4^+^ (B, C) or CD8^+^ (D, E) following stimulation with medium (B, D) or PMA plus ionomycin (C, E) are shown. The proportions (F) and numbers (G) of PMA plus ionomycin-stimulated CD4^−^ cells expressing IFNγ in the DLN of individual mice from the indicated groups (PBS, *n* = 6; OVA + IgG, *n* = 9; OVA + ES-62 + IgG, *n* = 10; OVA + anti-IFNγ, *n* = 10 and OVA + ES-62 + anti-IFNγ, *n* = 9; pooled from two independent experiments) are shown where the bar represents the mean of the group and the OVA + ES-62 + IgG group demonstrates significantly elevated levels of IFNγ production relative to the PBS control group (where ^∗^*P* ⩽ 0.05). (H) The proportions of PMA plus ionomycin-stimulated CD4^+^ cells expressing IFNγ in the DLN of individual mice from the indicated groups (PBS, *n* = 6; OVA + IgG, *n* = 9; OVA + ES-62 + IgG, *n* = 10; OVA + anti-IFNγ, *n* = 10 and OVA + ES-62 + anti-IFNγ, *n* = 9; pooled from two independent experiments) are shown where the bar represents the mean of the group and no significant differences are detected.

To address whether the ES-62 mediated stimulation of IFNγ production played a role in the protection afforded by the helminth product by resetting the Th1/Th2 balance, the effect of neutralising anti-IFNγ antibodies (or control IgG) during OVA-induced airway inflammation was investigated in two further independent experiments. It was noted (Fig. 4A) that a significantly greater proportion of PMA plus ionomycin-stimulated DLN cells from the OVA + ES-62, but not OVA, group treated with the control IgG expressed IFNγ than those from the PBS control group. However, this was not found to be the case in the presence of the neutralising anti-IFNγ antibodies (Fig. 4A). The expanded population of IFNγ-producing cells observed in the OVA + ES-62 mice (Fig. 3I, J) reflected increases in both IFNγ-producing CD4^+^ (Fig. 4B, C) and CD8^+^ (Fig. 4D, E) T cell populations. Analysis of the data from the two neutralising antibody studies showed that whilst the proportion (Fig. 4F) and number (Fig. 4G) of PMA plus ionomycin-stimulated CD4^−^ DLN cells (predominantly CD8^+^ T cells) producing IFNγ were significantly higher in the IgG-treated OVA + ES-62, but not OVA, group relative to the PBS control group, this effect was abolished by exposure to the neutralising anti-IFNγ antibodies. However, the proportion (Fig. 4H) of CD4^+^ DLN cells expressing IFNγ following stimulation with PMA plus ionomycin was not significantly different in the IgG-treated OVA + ES-62 group, relative to the OVA group, suggesting that the immunomodulatory IFNγ elicited by ES-62 was predominantly derived from CD4^−^ DLN cells, which are likely to be Tbet^+^CD8^+^ T cells (Szabo et al., 2002). Collectively, these data indicate that the neutralising anti-IFNγ antibodies were effective in vivo and suggest that blocking of this cytokine abrogates the resetting of the Th1/Th2 phenotype observed in OVA + ES-62 mice.

**Fig. 5:**
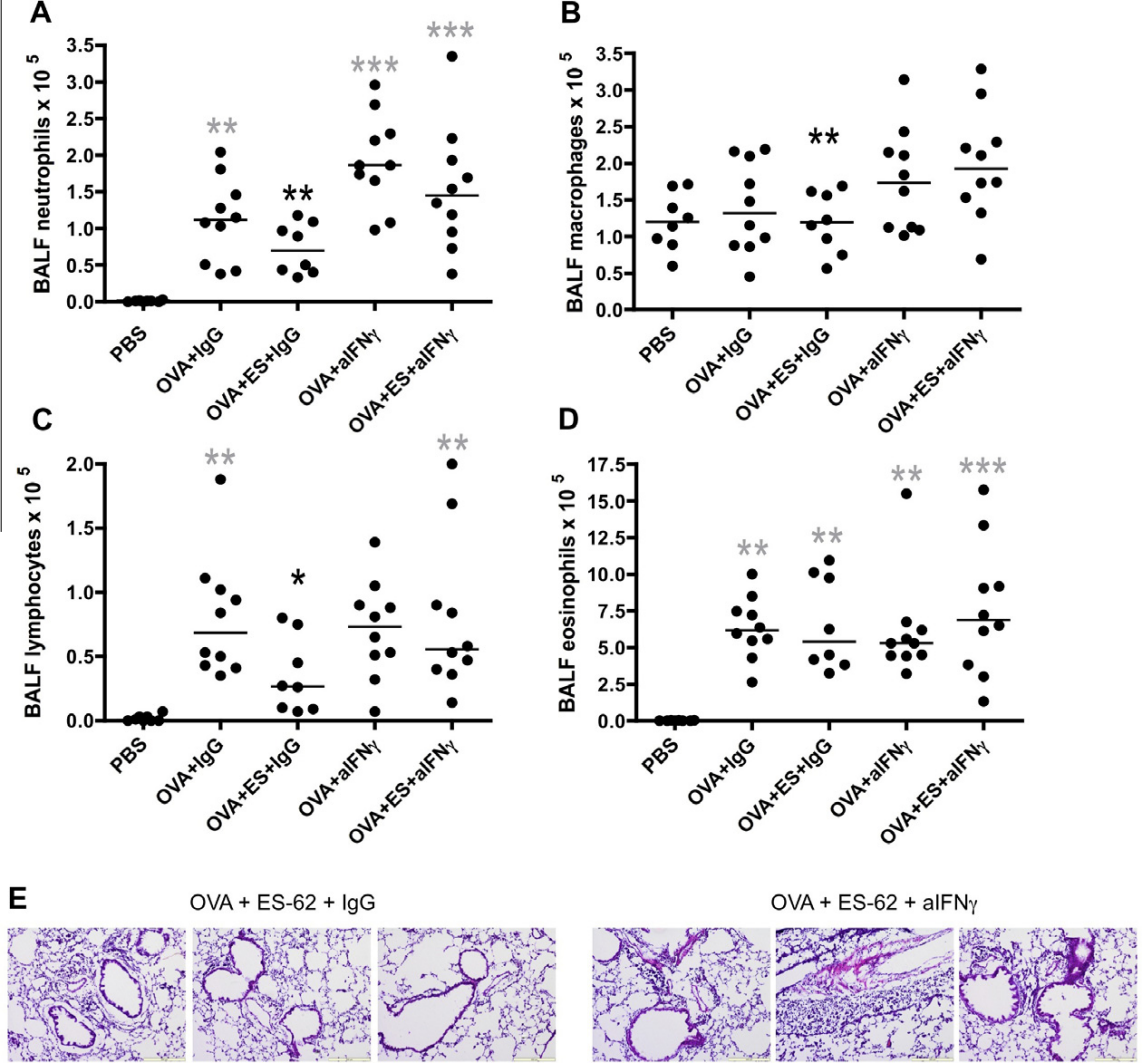
Neutralising anti-IFNγ antibodies prevent ES-62-mediated protection against cellular infiltration in murine ovalbumin (OVA)-induced airway inflammation. Differential neutrophil (A), macrophage (B), lymphocyte (C) and eosinophil (D) bronchoalveolar lavage fluid (BALF) cell counts for individual mice in each of the indicated treatment groups (PBS, *n* = 8; OVA + IgG, *n* = 10; OVA + ES-62 + IgG, *n* = 8; OVA + anti-IFNγ, *n* = 10 and OVA + ES-62 + anti-IFNγ, *n* = 10; pooled from two independent experiments) are shown where the bar represents the median of the group and black ^∗^*P* ⩽ 0.05 and ^∗∗^*P* ⩽ 0.01 are for OVA + ES-62 + IgG compared with OVA + ES-62 + anti-IFNγ groups whilst grey ^∗∗^*P* ⩽ 0.01 and ^∗∗∗^*P* ⩽ 0.001 are for PBS compared with the indicated treatment group. (E) Representative H & E sections from the OVA + ES-62 + IgG (*n* = 3 mice) and the OVA + ES-62 + anti-IFNγ (*n* = 3 mice) groups. Images were captured using an Olympus BX41 microscope mounted with an Olympus U-CMAD3 camera and Cell-B2.0 software. Images are at 10× magnification and include automatic yellow scale bar (200 μm) at the bottom right corner of image.

Importantly, therefore, and supportive of a role for ES-62 stimulated IFNγ in protecting against OVA-induced airway inflammation, the OVA + ES-62 group of mice treated with neutralising anti-IFNγ antibodies showed elevated levels of neutrophil (Fig. 5A), macrophage (Fig. 5B) and lymphocyte (Fig. 5C) infiltration of the BALF relative to those treated with control IgG. Moreover, although it did not reach statistical significance, the level of esoinophils also increased (Fig. 5D). In addition, analysis of lung pathology revealed higher levels of cellular infiltration and airway remodelling in the OVA + ES-62 group treated with the neutralising antibody, relative to the isotype control group (Fig. 5E).

**Fig. 6:**
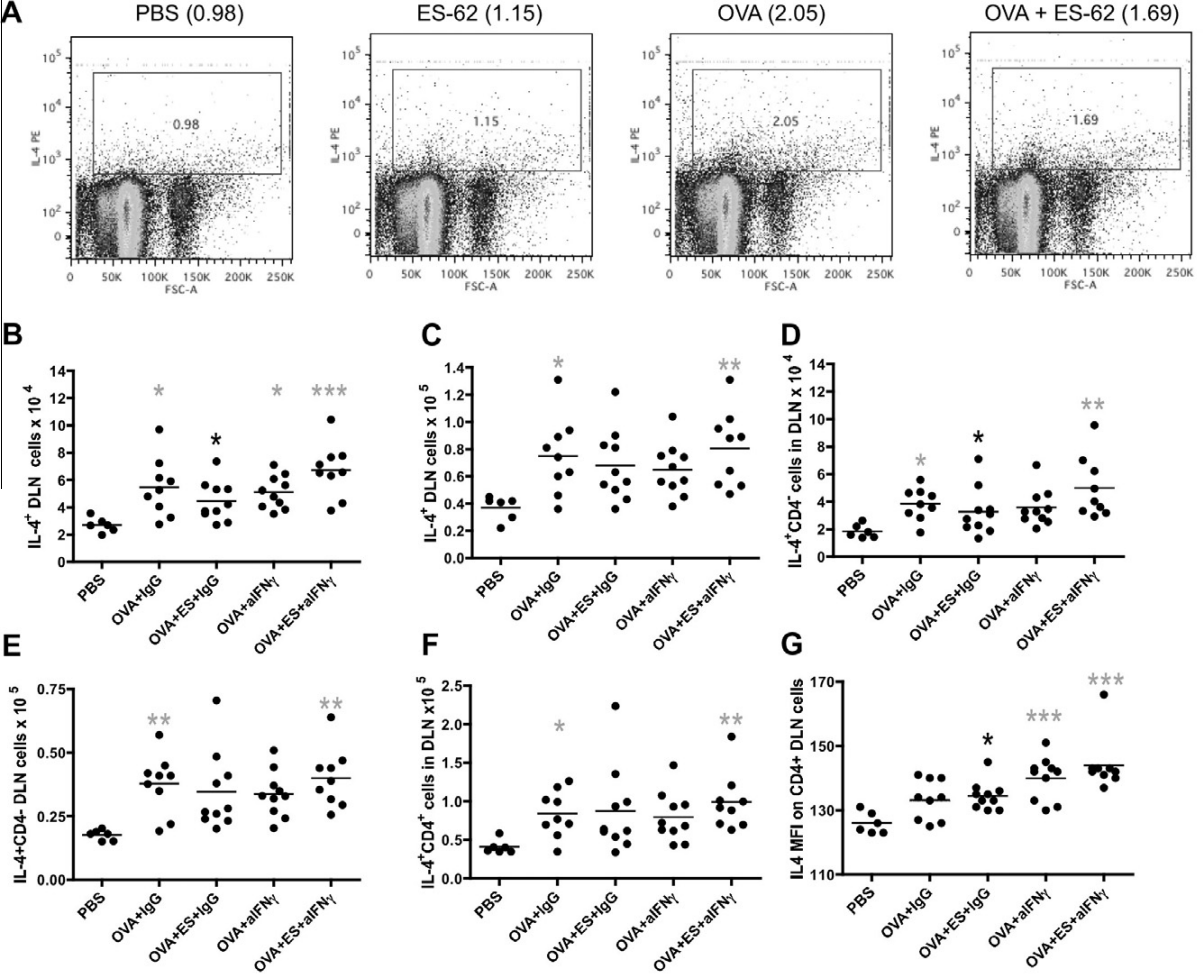
Neutralising anti-IFNγ antibodies modulate IL-4 responses in ES-62-treated mice undergoing ovalbumin (OVA)-induced airway inflammation. Draining lymph node (DLN) cells from the four indicated treatment groups were cultured with phorbol 12-myristate 13-acetate (PMA) plus ionomycin in the presence of Brefeldin A before staining for intracellular expression of IL-4 prior to FACS analysis: cytokine expression (*y*-axis) was plotted against side scatter (*x*-axis) and the percentage of DLN cells in the cytokine positive gate shown and annotated to the right of the plot labels (A). Data pooled from two further independent experiments show the numbers (B–F) and Mean Fluorescence Intensity (MFI) (G) of spontaneously- (B, D, G) and PMA plus ionomycin-stimulated (C, E, F) IL-4-producing DLN (B, C), CD4^−^ DLN (D, E) and CD4^+^ DLN (F, G) cells. The bar represents the mean value of the group where black ^∗^*P* ⩽ 0.05 represents the difference between the OVA + ES-62 + IgG compared with the OVA + ES-62 + anti-IFNγ group whilst grey ^∗^*P* ⩽ 0.05, ^∗∗^*P* ⩽ 0.01 and ^∗∗∗^*P* ⩽ 0.001 are for PBS compared with indicated treatment group (PBS, *n* = 6; OVA + IgG, *n* = 9; OVA + ES-62 + IgG, *n* = 10; OVA + anti-IFNγ, *n* = 10 and OVA + ES-62 + anti-IFNγ, *n* = 9; pooled from two independent experiments).

It appeared that the effect generated by anti-IFNγ treatment reflected, at least in part, that this cytokine does indeed act to reset the Th1/Th2 balance as although, as expected, ES-62-treated mice induced more IL-4-producing DLN cells than those of the control PBS group, the OVA + ES-62 group exhibited a lower proportion of IL-4-producing DLN cells than of the OVA group (Fig. 6A) in response to stimulation with PMA plus ionomycin. Moreover, analysis of data for individual mice from the two neutralising antibody experiments revealed that whilst the number of spontaneously- (Fig. 6B) and PMA plus ionomycin-stimulated (Fig. 6C) IL-4-producing DLN cells was significantly elevated in the OVA + IgG, but not OVA + ES-62 + IgG, group relative to the control PBS mice, in the presence of neutralising anti-IFNγ antibodies the OVA + ES-62 group exhibited significantly higher numbers of such cells (Fig. 6B, C). A similar pattern of IL-4 production was shown for both CD4^−^ (Fig. 6D, spontaneous; Fig. 6E, PMA plus ionomycin) and CD4^+^ T cells (Fig. 6F, PMA plus ionomycin), the latter subtype spontaneously expressing IL-4 at significantly higher levels in response to anti-IFNγ treatment (Fig. 6G), than to treatment with control IgG.

### 3.6 ES-62-mediated resetting of the Th1/Th2 balance in OVA-induced airway inflammation is associated with suppression of Th17/IL-17 responses

**Fig. 7:**
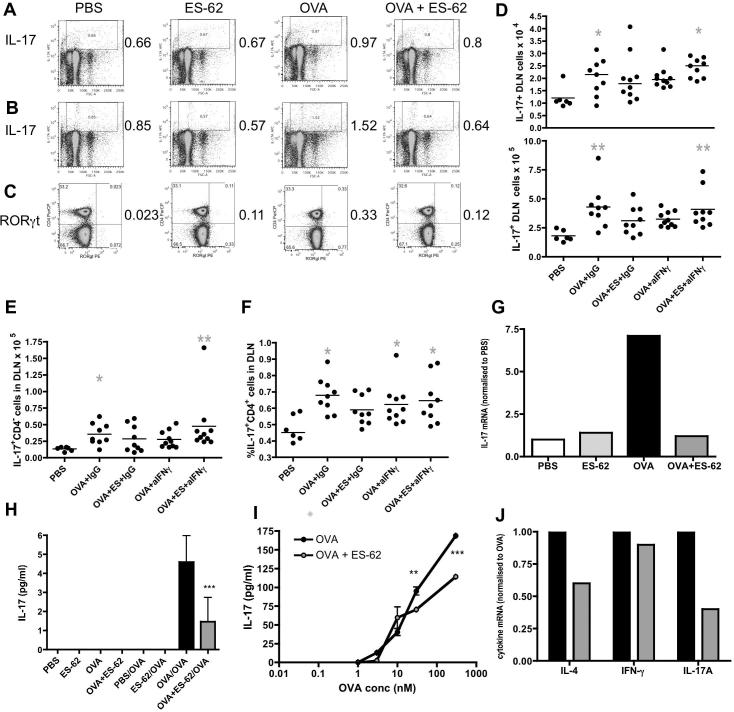
The phosphorylcholine-containing glycoprotein, ES-62, suppresses IL-17 responses in murine ovalbumin (OVA)-induced airway inflammation. Pooled draining lymph node (DLN) cells from the four indicated treatment groups were cultured with medium (A) or phorbol 12-myristate 13-acetate (PMA) plus ionomycin (B) in the presence of Brefeldin A before staining for intracellular expression of IL-17 prior to FACS analysis: cytokine expression (*y*-axis) was plotted against side scatter (*x*-axis) and the percentage of DLN in the cytokine positive gate shown and annotated to the right of the plots. (C) RORγt expressing unstimulated CD4^+^ T cells from the DLN of pooled mice from the four treatment groups are shown. The numbers (D) of spontaneously (upper; (PBS*, n* = 6; OVA + IgG, *n* = 9; OVA + ES-62 + IgG, *n* = 10; OVA + anti-IFNγ, *n* = 10 and OVA + ES-62 + anti-IFNγ, *n* = 9; pooled from two independent experiments)) and PMA plus ionomycin-stimulated (lower; (PBS, *n* = 6; OVA + IgG, *n* = 9; OVA + ES-62 + IgG, *n* = 9; OVA + anti-IFNγ, *n* = 10 and OVA + ES-62 + anti-IFNγ, *n* = 9; pooled from two independent experiments)) IL-17-producing DLN cells and the numbers of IL-17-producing CD4^−^ DLN cells (E) and proportions of IL-17-producing CD4^+^ cells (F) from PMA plus ionomycin-stimulated DLN cells from individual mice (PBS, *n* = 6; OVA + IgG, *n* = 9; OVA + ES-62 + IgG, *n* = 9; OVA + anti-IFNγ, *n* = 10 and OVA + ES-62 + anti-IFNγ, *n* = 9; pooled from two independent experiments) are presented; the bar represents the mean value of the group and grey ^∗^*P* ⩽ 0.05 shows the difference between the PBS and indicated treatment group. In an independent experiment (G), the levels of IL-17 mRNA (against a glyceraldehyde 3-phosphate dehydrogenase (GAPDH) reference) in the pooled DLN cells from the four indicated treatment groups are shown, normalised to the levels found in the control PBS group. (H) The levels of IL-17 detected by Luminex following stimulation of pooled DLN cells from mice in each group with medium or OVA (500 μg/ml) in a further independent experiment are shown and where ^∗∗∗^*P* ⩽ 0.001 for triplicate samples. In (I), pooled bone marrow-derived dendritic cells (bmDCs) from OVA and OVA + ES-62 mice were pulsed with OVA peptide on day 7 and cocultured with DO11.10 Tg CD4^+^CD62L^+^ T cells for 72 h before analysing the levels of IL-17 production in culture supernatants by ELISA. The data are presented as the mean ± S.D. where ^∗∗^*P* ⩽ 0.01, OVA compared with OVA + ES-62 at 30 nM of OVA peptide and ^∗∗∗^*P* ⩽ 0.001, OVA compared with OVA + ES-62 at 300 nm of OVA peptide in one experiment. In a further independent experiment, (J), the levels of IL-4, IFNγ and IL-17 mRNA (against a GAPDH reference) in the lungs from the OVA + ES-62 group (grey bars) are shown, normalised to the levels found in the OVA group (black bars).

Members of the IL-17 family of cytokines, including IL-17 (IL-17A and IL-17F) and IL-25 (IL-17E) have recently been reported to play key roles in promoting Th2 polarisation and eosinophil (Dias and Banerjee, 2012; Murdock et al., 2012), neutrophil (Iwakura et al., 2011) and macrophage recruitment (Barin et al., 2012) leading to AHR (Hellings et al., 2003; Linden, 2003; Kawaguchi et al., 2004; Schnyder-Candrian et al., 2006; Pichavant et al., 2008; Traves and Donnelly, 2008; Wang and Liu, 2008; Lajoie et al., 2010; Wang et al., 2010; Iwakura et al., 2011). In addition, Th17 cells have been shown to counter-regulate Th1 responses (Durrant and Metzger, 2010; Jager and Kuchroo, 2010). Thus it was investigated whether ES-62-mediated suppression of pathological Th2- and induction of counter-regulatory IFNγ-responses correlated with targeting of IL-17 production (Fig. 7). Analysis of the small populations of IL-17-producing DLN cells indicated that whilst those from the PBS, ES-62 and OVA + ES-62 groups exhibited comparable proportions of DLN cells spontaneously producing IL-17, this was slightly elevated in the OVA group (Fig. 7A). Similarly, the OVA mice exhibited the highest levels of such cells in response to PMA plus ionomycin but this population was substantially reduced in the OVA mice exposed to ES-62 in vivo (Fig. 7B). Such suppression reflected reductions in the proportions of IL-17-producing γδ, CD4 and CD8 T cells and RORγt-expressing DLN cells, including RORγt^+^CD4^+^ T cells (Fig. 7C and results not shown). Indeed, the data from the two neutralising antibody experiments showed that whilst mice from the OVA, but not OVA + ES-62, group treated with control IgG exhibited a higher number of DLN cells producing IL-17 than the PBS group, either spontaneously (Fig. 7D, upper graph) or following stimulation with PMA plus ionomycin (Fig. 7D, lower graph), the OVA + ES-62 group treated with neutralising anti-IFNγ antibodies showed significantly upregulated responses. Likewise, whilst mice from the OVA, but not OVA + ES-62, group treated with control IgG exhibited higher numbers of CD4^−^ (Fig. 7E) and percentages of Th17 (IL-17^+^CD4^+^, Fig. 7F) cells following stimulation with PMA plus ionomycin than the PBS control group, the OVA + ES-62 group treated with neutralising anti-IFNγ antibodies again showed significantly upregulated responses (Fig. 7E, F). Reflecting this, analysis of IL-17 mRNA production by DLN cells in an independent experiment revealed that in vivo exposure to ES-62 reduced the levels found in the OVA group to those comparable with levels of IL-17 mRNA found in the PBS or ES-62 groups (Fig. 7G) and although the levels observed were very low, this was reflected by reduced levels of OVA-specific IL-17 recall responses by DLN cells ex vivo in a further separate experiment (Fig. 7H). Such suppression of Th17 responses reflected, at least in part, a reduction in the ability of mice to prime Th17 responses as bmDCs from OVA + ES-62 mice induced significantly reduced OVA-specific Th17 responses relative to those from the OVA group (Fig. 7I). This suppression of IL-17 responses is not restricted to the DLN cells but is also apparent at the site of inflammation as qRT-PCR analysis indicated reductions in the levels of IL-4 and IL-17, but not IFNγ, mRNA in the lungs of mice from the OVA + ES-62, relative to the OVA, group of mice (Fig. 7J) in a further independent experiment.

Collectively therefore, the results outlined above provide support for the proposal that ES-62 is acting to suppress Th17/Th2-associated airway inflammation and consequent pathology, not by inducing Treg cells but by inducing counter-regulatory IFNγ responses to reset the Th1/Th2/Th17 balance.

## 4 Discussion

It was previously shown that prophylactic exposure to ES-62, a PC-containing glycoprotein secreted by the filarial nematode *A. viteae*, suppresses OVA-induced allergic airway disease in the mouse (Melendez et al., 2007). Although this protection was associated with desensitisation of mast cell responses, ES-62 was also shown to suppress the Th2 phenotype of the allergic response, as measured by a reduction in eosinophilia and IL-4 levels in the lungs. We now show that ES-62 suppresses the lung infiltration by a heterogeneous population of cells, particularly neutrophils, a phenotype that is associated with severe asthma and is generally refractory to conventional anti-inflammatory therapies, including steroids (Ito et al., 2008; Iwakura et al., 2011; Yang et al., 2012). Consistent with the observed inhibition of cells infiltrating the lungs, we also found that exposure to ES-62 suppressed IL-17 responses, which have recently been shown to play key roles in recruitment of eosinophils, neutrophils and macrophages as well as in the promotion of Th2 polarised inflammation and development of severe asthma (Lajoie et al., 2010; Wang et al., 2010; Iwakura et al., 2011; Barin et al., 2012; Dias and Banerjee, 2012). Such protection against Th2/Th17-associated airway inflammation did not reflect induction of Treg responses (no increased IL-10 or Foxp3 expression) but rather a switch in priming towards increased Tbet expression and IFNγ production. Further support for ES-62 suppressing pathogenic Th2/Th17 responses by inducing counter-regulatory IFNγ responses (Durrant and Metzger, 2010; Jager and Kuchroo, 2010) was provided by our studies showing that neutralising anti-IFNγ antibodies blocked the protection against airway inflammation afforded by ES-62, in terms of cellular infiltration of the lungs as well as airway remodelling, and that this loss of protection was accompanied by elevated Th2 and Th17 responses.

The anti-inflammatory mechanism that is employed by ES-62 was compared with that associated with some other helminths and their products. It appears to be quite distinct from the approach utilised by the gastrointestinal (GI) nematode, *Heligmosomoides polygyrus*, which can inhibit OVA-induced airway inflammation in mice via the induction of a Treg cell population (Wilson et al., 2005). Moreover, there are a number of differences observed between the immunomodulatory effects of an *H. polygyrus* infection and ES-62 when affording protection. For example, the GI nematode only inhibited production of Th2 effector cytokines (IL-5/IL-13) whereas ES-62 also had an inhibitory effect on a cytokine that facilitates Th2 responses, IL-4. This is likely to explain why, unlike in the *H. polygyrus* study, we observed a decrease in IgE levels. We also could not find any evidence of increased levels of Treg cells as evidenced by Foxp3 expression or production of IL-10 in DLN cell cultures from ES-62 exposed animals although this regulatory cytokine is also not the principal facilitator of the protective effects of *H. polygyrus* (Wilson et al., 2005). It is worth emphasising at this point that our present data are consistent with our previous work indicating that there is little evidence that ES-62 induces any form of Treg population (Marshall et al., 2005). In agreement with our observations, recent data (McSorley et al., 2012) reported a lack of expansion of Tregs upon treatment of allergic mice with *H. polygyrus* excretory–secretory (HES) products, even though administration of HES products at the sensitisation stage significantly diminished numbers of eosinophils and neutrophils in BAL and macrophages in the lung. In this respect therefore, the effects of HES products are similar to those afforded by ES-62. Importantly, however, treatment with HES products abolished OVA-induced IFNγ, in addition to downregulating IL-4, IL-5 and IL-13 production, a finding which appears to rule out the possibility that HES and ES-62 might share a common mechanism of action in suppression of allergic airway inflammation.

The immunomodulatory effects being observed with ES-62 are also comparable to those described when AHR to OVA was inhibited by an extract of the GI nematode, *Ascaris suum* (Lima et al., 2002). Here, each of IL-4 and IL-5 in BALF, and IgG1 and IgE in serum was reduced. As with ES-62 (Melendez et al., 2007), airway hyper-reactivity to methacholine was also inhibited in this model system (Lima et al., 2002). One difference from the *A. suum* study was that we did not see a reduction in serum IgG1 but we previously showed a lack of a relationship between the ability of ES-62 to induce IL-4 and promote IgG1 responses to OVA (Marshall et al., 2005). A decrease in IgE without a decrease in IgG1 could perhaps reflect the activity of IL-10 (Jeannin et al., 1998) but as mentioned above we could find no evidence for increased IL-10 in our study. More recently, another filarial nematode-derived molecule, cystatin, has been found to protect against OVA-induced airway hyper-reactivity, showing many of the properties of ES-62 in reducing eosinophilia, IgE and IL-4 (Schnoeller et al., 2008). Although similar to ES-62, the effect of this molecule does not appear to be due to induction of Tregs, it differs from ES-62 in relying on IL-10 for some activities. In particular, whilst a role for IL-10-producing macrophages is reported in the cystatin study (Schnoeller et al., 2008), we have found that the spontaneous secretion of IL-10 from macrophages derived from mice with airway disease was reduced in the group exposed to ES-6*2* (results not shown). We also found IL-10 responses to be suppressed by ES-62 throughout this current study and although such effects are perhaps initially surprising, recent findings suggesting that IL-4 and IL-10 are essential for pulmonary arterial remodelling in response to inhaled *Aspergillus* allergen in a mixed Th1/Th2/Th17 microenvironment (Shreiner et al., 2012) may suggest that this suppression of IL-10 contributes to the protection afforded by the helminth product.

Whilst the immunoregulatory mechanisms involved have not been fully delineated, as mentioned earlier, ES-62 appears to act, at least in part, by resetting the Th1/Th2/Th17 balance by inducing a switch in priming towards a Th1-like phenotype (relative increase in IFNγ and induction of Tbet expression, involving both CD4^+^ and CD8^+^ T cells). Whilst Tbet-expressing induced Tregs have been reported (Anderson et al., 2006; Cobbold, 2006), it is unlikely that the induction of Tbet expression by ES-62 reflects the generation of such induced Tregs as these have also been reported to mediate their effects, at least in part, via IL-10 production which is, as noted earlier, reduced in the ES-62-treated groups. Moreover, recent analysis of Tbet^−/−^ mice has revealed that dysfunction of Tbet may be involved in the pathogenesis of allergic airway disease and that Tbet^+^CD4^+^ cells can inhibit Th2-mediated eosinophilia (Fujiwara et al., 2007). Consistent with this, expression levels of Tbet appear to be reduced in the airways of asthmatic patients (Finotto et al., 2002) and polymorphisms in this transcription factor have been associated with airway inflammation and hyper-responsiveness (Raby et al., 2006; Munthe-Kaas et al., 2008; Durrant and Metzger, 2010). Collectively, therefore, these findings provide a potential rationale for how resetting of the Th1/Th2 balance associated with increased Tbet and IFNγ expression by ES-62 can suppress airway inflammation.

Given that Th17 cells can counter-regulate Th1 development and that IL-17 family members (IL-17A, F and IL-25 (IL-17E) have been shown to promote allergic airway inflammation (Linden, 2003; Kawaguchi et al., 2004; Traves and Donnelly, 2008; Wang and Liu, 2008; Durrant and Metzger, 2010; Jager and Kuchroo, 2010), a potential mechanism for the induction of counter-regulatory IFNγ production is suggested by our findings that the ES-62-driven switch in the Th1/Th2 balance was accompanied by both a decrease in the proportion of DLN cells producing IL-17 and in the ability of bmDCs from ES-62-treated mice to prime Th17 responses. Intriguingly, we have recently demonstrated that the ability of ES-62 to protect against IL-17-dependent Th1-associated autoimmune inflammation in the collagen-induced mouse model of arthritis reflected targeting of a complex (γδ T cell, DC and Th17 interactions) cellular network in which ES-62 suppressed both initiation of, and maintenance of ongoing, IL-17 responses (Pineda et al., 2012). Indeed, the IL-17-producing CD4^−^ DLN cell population targeted by ES-62 in this present study may include a similar population of IL-17-producing γδ T cells modulated by the helminth product.

Thus, ES-62 may target IL-17 as a mechanism to suppress either Th1 or Th2 pathology by resetting the Th1/Th2 balance. Support for this proposal in terms of Th2 allergic airway inflammation is provided by studies demonstrating that the extent of AHR following exposure to methacholine correlates with the levels of IL-17 (Barczyk et al., 2003; Kolls et al., 2003; Durrant and Metzger, 2010) and consistent with this, we have found that exposure to ES-62 in vivo substantially suppresses AHR (Melendez et al., 2007). Moreover, studies on IL-17A- or IL-17RA-deficient mice showed decreased induction of OVA-specific T cells, reduced eosinophilic inflammation and lower levels of serum IgE (Nakae et al., 2002; Schnyder-Candrian et al., 2006; Durrant et al., 2009; Durrant and Metzger, 2010) and may reflect our findings that treatment of the OVA + ES-62 group with neutralising anti-IFNγ antibodies results in elevated levels of Th2 cells (perhaps reflecting the increase in lymphocyte infiltration of the BALF) as well as the increased neutrophil and macrophage recruitment more normally associated with elevated Th17 responses. Of particular interest, therefore, given our findings with respect to ES-62-mediated induction of Tbet and resetting of the Th1/Th2 balance, OVA-induced airway inflammation is predominantly Th17-mediated in Tbet-deficient animals and this inflammation can be suppressed by treatment with IL-12 during the challenge phase (Durrant et al., 2009; Durrant and Metzger, 2010).

However, it should be noted that IL-17 may also act in sensitised mice to dampen the allergic response by inhibiting chemokine production, eosinophilia and bronchial hyperactivity (Hellings et al., 2003; Schnyder-Candrian et al., 2006) and that human hyper-IgE syndrome is associated with a failure in IL-17 production by T cells (Ma et al., 2008; Milner et al., 2008). Moreover, IFNγ-producing Th1 cells have been implicated in the development of severe macrophage-dependent, steroid-resistant asthma (Yang et al., 2009). However, these pathogenic effects of IFNγ are entirely dependent on crosstalk with endotoxin-driven TLR4/MyD88 pro-inflammatory signalling and we previously showed that ES-62 subverts TLR4/MyD88 (and TLR2/TLR9 but not TLR3 which is MyD88 independent) signalling to an anti-inflammatory phenotype and suppresses MyD88 expression during Th17 development (Goodridge et al., 2005; Melendez et al., 2007; Pineda et al., 2012). Taking these findings into account, it seems likely that both IFNγ and IL-17 might exhibit dual pathogenic and protective roles depending on the temporal and microenvironmental context of the response.

In conclusion, the finding that ES-62 acted to suppress Th2 responses of DLNs to heterologous Ag is consistent with the idea that, despite biasing immune responses towards a Th2-like phenotype (Harnett and Harnett, 2010), nematode infections may protect against atopy. Thus, although ES-62 may have a tendency to promote a weak Th2-like immunological phenotype in naïve mice (Houston et al., 2000; Whelan et al., 2000; Marshall et al., 2005), this does not prevent it inhibiting Th2 responses when they are contributing to pathological inflammation. ES-62 appears to act to reset the Th1/Th2 balance towards a more “neutral” or anti-pathologic inflammatory phenotype by targeting Th17/IL-17. Interestingly, this resetting of the Th1/Th2 balance is Ag-specific as mitogen-induced responses remained unaltered (results not shown and McInnes et al., 2003): thus, ES-62 can act to limit Th1 or Th2 pathology induced by chronic inflammation whilst allowing the generation of necessary immune responses to remain intact. Collectively, these studies indicate that ES-62 or, more likely, small molecule analogues (patent application number 1214106.5) may have therapeutic potential in asthma, particularly for those subtypes of patients (e.g. smokers, steroid resistant) who are refractory to current treatments.
